# Leiomyosarcoma of uterus in pregnancy: A case report

**DOI:** 10.1097/MD.0000000000048608

**Published:** 2026-05-08

**Authors:** Min Lin, Chunjing Chen, Xiaoxiao Wu, Huanming Lin, Yulan Lin, Jiawen Lin

**Affiliations:** a Gynaecology, Sanming First Hospital Affiliated to Fujian Medical University, Sanming, China.

**Keywords:** autologous blood transfusion, Misdiagnosis, pregnancy, uterine leiomyosarcoma

## Abstract

**Rationale:**

Differentiation between uterine leiomyoma and leiomyosarcoma of uterus(LMS) is often challenging, especially in the context of pregnancy, which may delay subsequent diagnosis and treatment.

**Patient concerns:**

A 35-year-old pregnant woman was found to have uterine fibroids based on preand prenatal ultrasound findings, and she remained asymptomatic. Due to the large tumor size, which affected fetal delivery and incision closure, she underwent cesarean section combined with myomectomy. Postoperative pathology revealed LMS. Further CT scans indicated pulmonary metastases, leading to a diagnosis of stage IVB LMS. Subsequently, the patient underwent laparoscopic total hysterectomy with bilateral salpingo-oophorectomy, pelvic lymphadenectomy, peritoneal biopsies, and omental sampling. The patient remains under follow-up.

**Diagnoses:**

The diagnosis was made based on postoperative pathology and relevant imaging findings.

**Interventions:**

The patient was treated with surgery and chemotherapy.

**Outcomes:**

The patient underwent surgery and chemotherapy, and was still under follow-up.

**Lessons:**

Attention should be paid to the diagnosis of LMS in the context of pregnancy. When facing a fibroid of uncertain nature during cesarean section, it is necessary to assess whether myomectomy is feasible and whether intraoperative autologous blood transfusion can be performed. Therefore, timely diagnosis through comprehensive clinical and imaging findings is essential to avoid misdiagnosis and ensure prompt intervention.

## 1. Introduction

LMS is a rare and highly malignant tumor, and its occurrence during pregnancy is exceedingly uncommon. The disease typically presents with symptoms such as abdominal pain, palpable abdominal mass, irregular vaginal bleeding, and increased vaginal discharge.^[1]^ This case report describes a misdiagnosed case of LMS, initially identified as a leiomyoma during pregnancy and treated with chemotherapy. The patient is still being followed up. This case report has passed the ethical review of the Biomedical Research Ethics Committee of Fujian Medical University, and the patient’s consent has been obtained.

## 2. Case report

**Figure 1.**
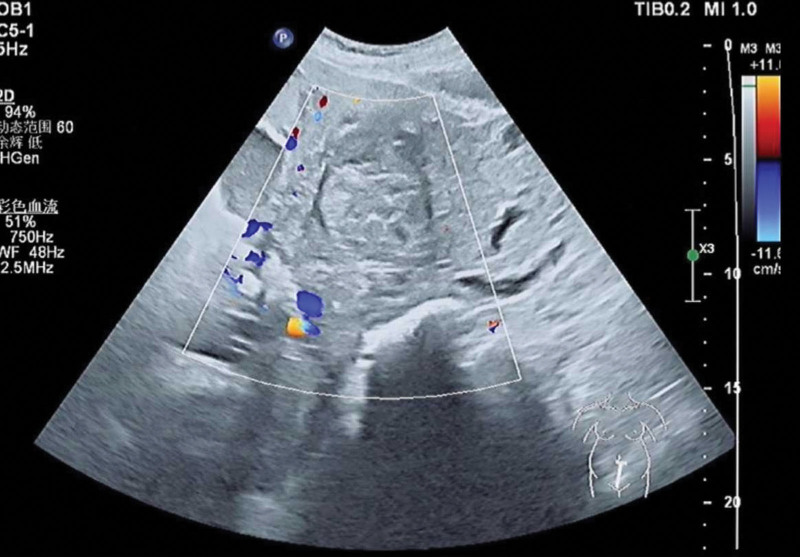
Obstetric ultrasound images about uterine leiomyoma.

**Figure 2.**
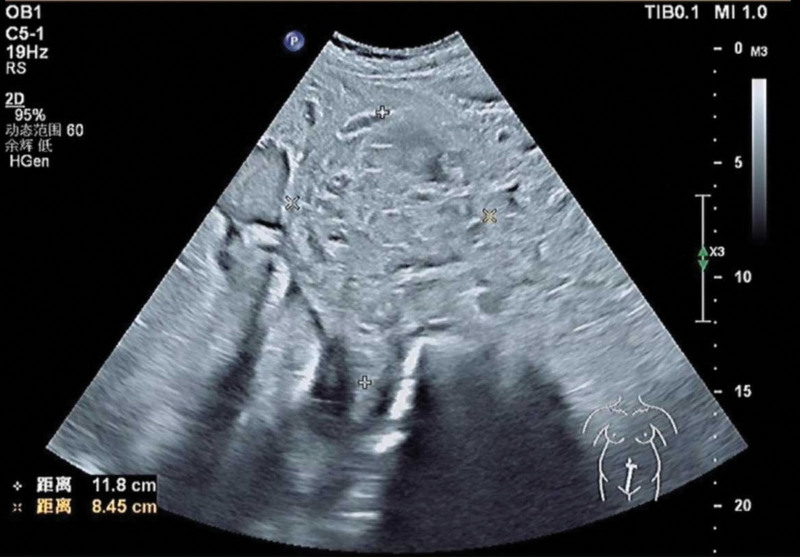
Obstetric ultrasound images about uterine leiomyoma.

**Figure 3.**
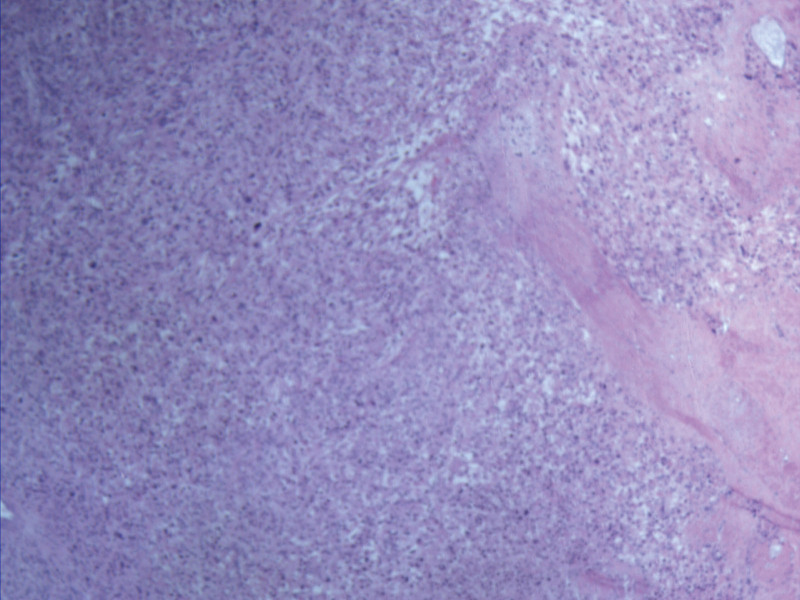
Pathological image following myomectomy of uterine fibroids.

A 35-year-old woman with a menstrual cycle of 7/28–32 days and moderate menstrual flow was admitted due to a lower abdominal mass discovered more than 2 years prior. No other significant medical history. In 2022, ultrasonography revealed a uterine leiomyoma measuring approximately 1.8 × 1.5 cm. Subsequently, no regular follow-up ultrasound examinations were performed. In May 2024, an early intrauterine pregnancy was confirmed, and the leiomyoma had enlarged to 6.0 × 5.2 × 6.3 cm. By December 2024, the mass had expanded to 11.8 × 8.5 cm, with no detectable color Doppler flow signals (shown in Figures 1 and 2). Due to preeclampsia and obstruction of fetal descent by the large leiomyoma, cesarean section was performed at 36 + 6 weeks of gestation. As the uterine fibroids were located in the middle and lower segments and could not be avoided, they affected the suturing of the uterine incision. Therefore, the uterine fibroids were removed together. A male neonate was delivered with Apgar scores of 10-10-10 and a birth weight of 3660 g. Intraoperative findings revealed a firm, 6 × 4 cm mass protruding from the anterior uterine wall toward the serosal surface, with tortuous vessels penetrating the uterine cavity, exhibiting a mushroom-like morphology and fish-flesh appearance upon sectioning. Due to significant bleeding after the delivery of the fetus and placenta, autologous blood transfusion was initiated after suctioning the amniotic fluid. Maternal blood was collected into a reservoir with anticoagulation, initially filtered through a centrifugation bowl to retain purified concentrated suspended red blood cells, and then transfused back to the mother via an intravenous line after passing through a leukocyte filter. Blunt enucleation of the mass resulted in approximately 1000 mL of blood loss, accompanied by hypotension and decreased oxygen saturation, necessitating the transfusion of 3 units of packed suspended red blood cells and bilateral ligation of the ascending branches of the uterine artery. The total volume of intraoperative autologous blood reinfusion was 600 mL. Histopathological examination revealed a spindle cell tumor with cellular atypia and focal necrosis. Immunohistochemistry (IHC) confirmed uterine leiomyosarcoma, showing diffuse moderate-to-severe atypia, tumor giant cells, >30 mitoses/10 high-power fields(HPF), pathological mitotic figures, coagulative necrosis, and vascular invasion. The tumor exhibited focal epithelioid and myxoid differentiations. IHC markers: Actin (+++), Caldesmon (+++), Desmin (+++), EMA (++), P16 (+++), FH (+++, intact), ER (focal +), PR (+), P53 (–, nonsense mutation), CD10 (focal +), Ki-67 (~70%+). The referral pathology consultation concurred(shown in Figure 3).

**Figure 4.**
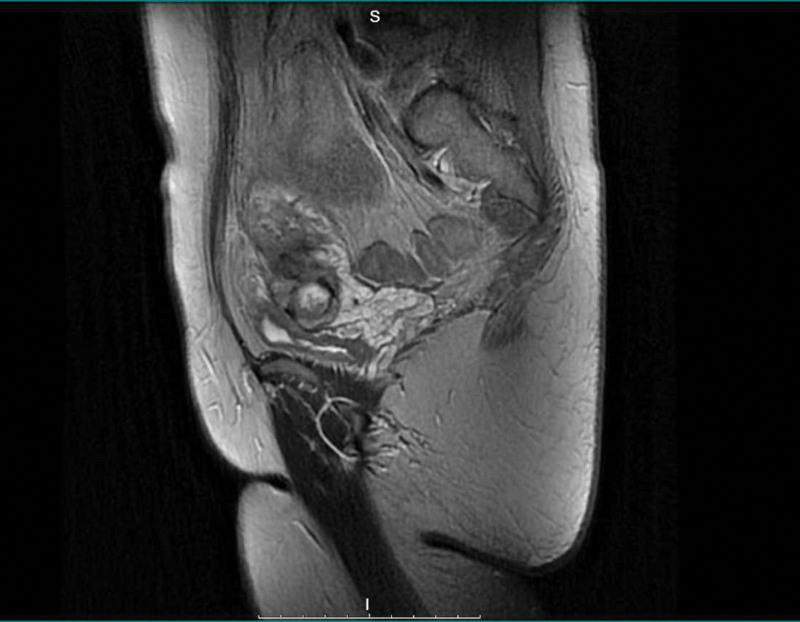
First postoperative MRI image.

**Figure 5.**
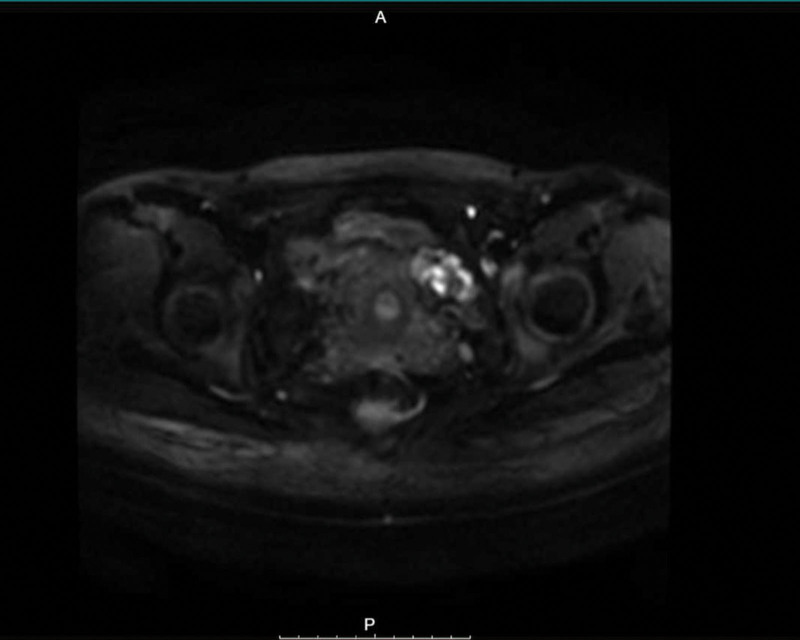
First postoperative MRI image.

**Figure 6.**
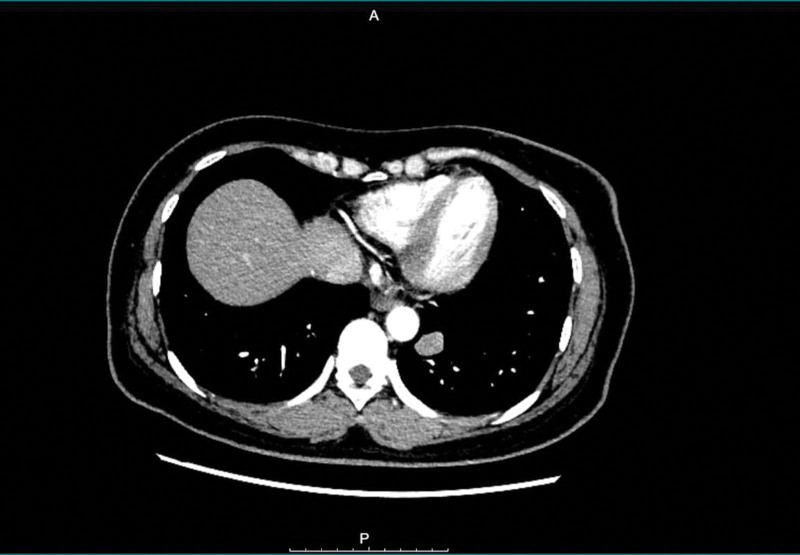
CT image indicates possible lung metastasis.

**Figure 7.**
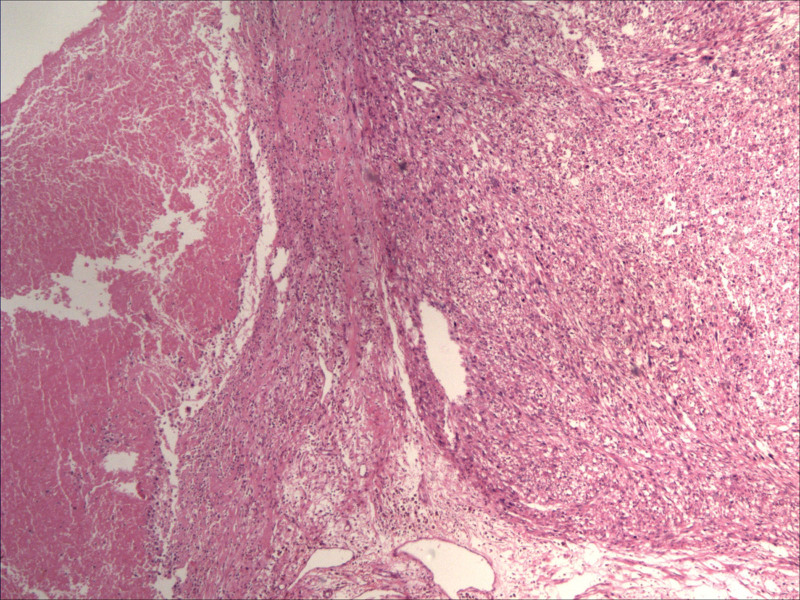
Pathological image after the second surgery.

**Figure 8.**
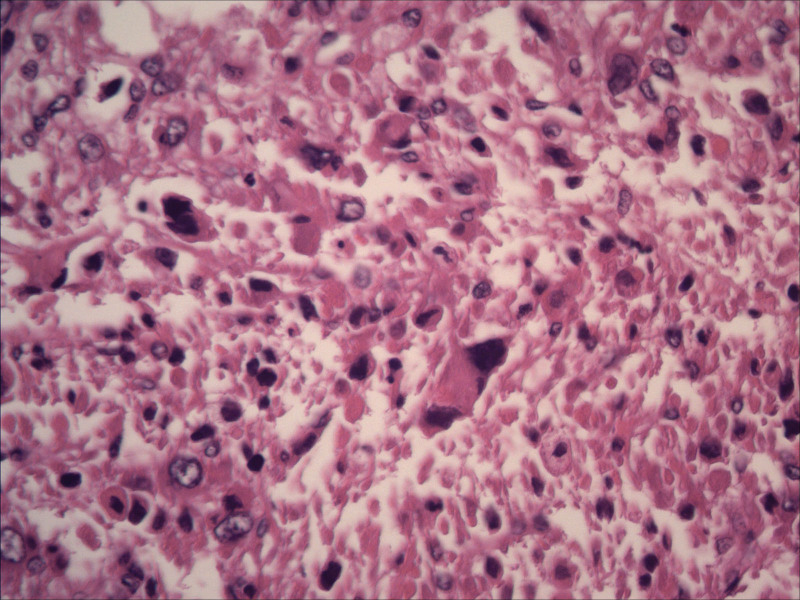
Pathological image after the second surgery.

Two months later, PET-CT and MRI showed a circular metabolically active mass in the lower segment of the uterus, which may have been a residual or recurrent LMS, and postoperative changes could not be ruled out. Slightly enlarged pelvic and iliac vessel lymph nodes were observed, indicating an increase in metabolism(shown in Figure 4 and 5). However, its nature remains unclear. Multiple pulmonary nodules with increased metabolism are suggestive of metastasis(shown in Figure 6). FIGO 2009 staging classified the disease as IVB. The patient underwent laparoscopic total hysterectomy with bilateral salpingo-oophorectomy, pelvic lymphadenectomy, peritoneal biopsies, and omental sampling. Intraoperatively, a 4 cm mass in the left uterine wall was noted without omental, peritoneal, liver, or splenic involvement. Searching for tumor cells in ascites without obvious abnormalities. Pathology confirmed LMS with focal vascular invasion, hemorrhage and necrosis, but no nodal (29 nodes examined) or distant metastases(shown in Figure 7 and 8).

Postoperatively, the patient reported no discomfort after surgery, and adjuvant chemotherapy was initiated in medical oncology(a total of 6 cycles). During follow-up, the patient reported no discomfort.

## 3. Discussion

The patient, initially diagnosed with a leiomyoma in 2022, exhibited rapid growth during pregnancy (doubling in size by the first trimester). Asymptomatic presentation and absence of Doppler flow contributed to the misdiagnosis. Hormonal surges during pregnancy, particularly estrogen and progesterone from placental synthesis, may accelerate tumor progression, as most LMS express these receptors.^[2]^ Histogenesis remains debated: nonleiomyoma of the uterus could be malignant because of hormonal action or LMS at first. Matsuo et al^[3]^ hypothesized that male fetuses might promote LMS growth via Y chromosome–related mechanisms, although this remains unproven. In this case, LMS may have preexisted and expanded rapidly due to hormonal and immunological alterations.^[4]^ The sonographic features of LMS typically include irregular borders and mean diameters > 8 cm. Misdiagnosis may reflect overlooked Doppler parameters or the effects of estrogen and progesterone on Doppler ultrasound blood flow signals.^[5]^

Challenges in Differentiating Uterine Leiomyomas from Sarcomas in the Context of Pregnancy: Similar Symptoms and Imaging Findings. Both conditions may present with pain and vaginal bleeding. Red degeneration (necrosis) of a leiomyoma during pregnancy often causes severe abdominal pain and fever, whereas LMS can also induce pain due to rapid growth and ischemic necrosis. Under the influence of elevated estrogen and hCG levels during pregnancy, benign leiomyomas may also enlarge rapidly, making it difficult to distinguish their growth rates from that of LMS. On imaging, leiomyomas in pregnancy frequently undergo degeneration, leading to irregular cystic areas and complex echogenicity on ultrasound or MRI, which can be easily confused with the necrotic foci of LMS. Additionally, uterine artery blood flow increases significantly during pregnancy, often resulting in rich vascularity around leiomyomas. This finding is challenging to differentiate from neovascularization within the LMS on ultrasound or contrast-enhanced MRI.

The focus of management varies across different stages. First, Preconception: The emphasis is on evaluating the location and size of the leiomyomas to plan for surgical feasibility. For submucosal leiomyomas and some large intramural leiomyomas that may interfere with embryo implantation or affect embryonic development, surgical intervention before pregnancy is recommended. Dynamic observation is acceptable for subserosal and most small intramural leiomyomas. In this patient, the presence of large uterine leiomyomas before pregnancy warranted surgical myomectomy to create a favorable environment for conception.

Intraoperative autologous transfusion was performed during the cesarean section. The detection of postoperative pulmonary metastases raised unresolved questions: Were the metastases pre, or did the transfusion potentiate hematogenous spread? Zhu et al^[6]^ found no evidence linking autologous transfusion to increased recurrence and metastasis in malignancy based on evidence-base research; however, theoretical risks persist for vascular-invasive solid tumor, which was likely higher capacity. These people did Autologous transfusion contributed to unfavorable outcomes, but no clinical evidence has been found to date.

Early diagnosis is critical given LMS’s poor prognosis (5-year survival: 20–30%). Based on the postoperative pathological type and staging, adjuvant chemoradiotherapy, hormonal treatment, or immunotherapy may improve outcomes in advanced cases. Multimodal therapy, including surgery and chemotherapy, is indicated in cases of distant metastasis.

## 4. Conclusion

Pregnancy-associated “leiomyomas” with rapid growth or irregular bleeding in the early trimester of pregnancy warrant suspicion of leiomyoma degeneration, including LMS.^[2]^ Serial ultrasound/MRI monitoring for suspected uterine leiomyosarcoma during pregnancy is essential. During the follow-up period, the clinical symptoms and signs of the patients were recorded. In addition, attention should be paid to myoma size. Highly suspicious cases may require pregnancy termination for timely intervention and prolonged survival.

## Author contributions

**Writing – original draft:** Min Lin.

**Writing – review & editing:** Chunjing Chen.

**Investigation:** Xiaoxiao Wu, Yulan Lin, Jiawen Lin.

**Supervision:** Huanming Lin.
